# Interictal Epileptiform Discharges and Alzheimer's Disease‐Associated Cortical Atrophy in Late‐Onset Epilepsy of Unknown Etiology

**DOI:** 10.1002/alz70861_108284

**Published:** 2025-12-23

**Authors:** L. Brian Hickman, Hsiang Yeh, Haley Willem, Bhavya Pandey, Mohit Bandla, Alexander Fish, Adnan Husein, Corinne Allas, Harika Kottakota, Lauren Herzog, Noriko Salamon, Daniel H Silverman, John M. Stern, Keith Vossel

**Affiliations:** ^1^ University of California, Los Angeles, Los Angeles, CA USA; ^2^ University of Pennsylvania, Philadelphia, PA USA

## Abstract

**Background:**

Late‐onset epilepsy of unknown etiology (LOEU) is associated with elevated dementia risk and may reflect early pathological changes from Alzheimer’s disease. We assessed for relationships between interictal epileptiform discharges on EEG, patterns of cerebral atrophy, and subsequent development of dementia in LOEU.

**Method:**

We retrospectively assessed neuroimaging data from 51 patients with LOEU, defined as seizure onset at ≥55 years excluding patients with pre‐existing dementia, cortical lesions, or hippocampal sclerosis. Dementia occurrence was determined from neurologist‐documented problem lists. Clinical brain MRIs with ≤1 mm isotropic voxel T1‐weighted scans were analyzed. Volumetric gray matter measurements of medial temporal and parietal regions were performed using FreeSurfer 7.4.1. EEG reports were available for 47 patients. Analyses were performed using multivariable regression, controlling for sex, age, scanner field strength, and log‐transformed hours of EEG recording.

**Result:**

The average age at MRI was 68.6 years (SD: 6.7) and 20 (39.2%) were female. Six patients (11.8%) were diagnosed with dementia after epilepsy onset during an average follow‐up period of 6.0 years. Patients with LOEU who developed dementia demonstrated significantly smaller left posterior cingulate cortex volumes compared to those without dementia (‐638.7 mm^3^, *p* < 0.01). Of the 47 patients with EEGs, 10 (21.3%) had isolated left, 5 (10.6%) had isolated right, and 4 (8.5%) had bilateral independent discharges (2 left‐predominant, 1 right‐predominant, and 1 equally bilateral). Epileptiform discharges were associated with lower volumes in right entorhinal (‐248.7 mm^3^, *p* < 0.02), right fusiform (‐806.0, *p* < 0.02), left fusiform (‐796.8 mm^3^, *p* < 0.03), and right precuneus (‐860.3 mm^3^, *p* < 0.03) cortices compared to LOEU without discharges. In subgroup analyses, patients with right hemispheric/right predominant epileptiform discharges had decreased right entorhinal cortex (‐469.1 mm^3^, *p* < 0.01), left parahippocampal gyrus (‐366.6 mm^3^, *p* < 0.02), and left posterior cingulate (‐554.0 mm^3^, *p* < 0.05) volumes compared to patients without epileptiform discharges.

**Conclusion:**

Patients with LOEU who develop dementia demonstrate decreased left posterior cingulate cortex volume. Interictal epileptiform discharges in LOEU are associated with bilateral medial temporal and parietal atrophy. Further work is needed to assess relationships between seizure onset location, atrophy patterns, and dementia risk in LOEU.

